# Mechanisms of hypha orientation of fungi

**DOI:** 10.1016/j.mib.2009.05.007

**Published:** 2009-08

**Authors:** Alexandra Brand, Neil AR Gow

**Affiliations:** Aberdeen Fungal Group, School of Medical Sciences, Institute of Medical Sciences, University of Aberdeen, Aberdeen AB25 2ZD, UK

## Abstract

Hypha orientation is an essential aspect of polarised growth and the morphogenesis, spatial ecology and pathogenesis of fungi. The ability to re-orient tip growth in response to environmental cues is critical for colony ramification, the penetration of diverse host tissues and the formation of mating structures. Recent studies have begun to describe the molecular machinery regulating hypha orientation. Calcium signalling, the polarisome Bud1-GTPase module and the Tea cell-end marker proteins of the microtubule cytoskeleton, along with specific kinesins and sterol-rich apical microdomains, are involved in hypha orientation. Mutations that affect these processes generate normal-shaped, growing hyphae that have either abnormal meandering trajectories or attenuated tropic responses. Hyphal tip orientation and tip extension are, therefore, distinct regulatory mechanisms that operate in parallel during filamentous growth, thereby allowing fungi to orchestrate their reproduction in relation to gradients of effectors in their environments.

## Introduction

Most fungi are sessile filamentous organisms that grow by extending the tips of hyphae to form an expanding mycelial network. Tip growth, therefore, represents a form of cellular motility and hyphae must be able to coordinate this motility by extending and orientating the trajectory of hyphal extension. This enables them to optimise their behaviour and make appropriate responses to environmental cues. For example, hyphae of mating gametes must be able to undergo sexual orientation that leads to cell fusion, karyogamy and meiosis; vegetative hyphae exhibit positive aerotropism and negative autotropism to enable hyphae in a colony to ramify into evenly dispersed mycelial networks that maximally exploit nutrients in the substratum. Hyphae navigate around impenetrable objects and dermatophytes intercalate between layers of cornified epithelium and other tissues. Hyphae can also fuse and follow complex morphogenetic programmes to generate structures such as perithecia, lichenous thalli and the sporocarps of mushrooms. Therefore, the ability to orient the axis of growth of the hyphae within a mycelium is vital to the saprophytic, symbiotic and parasitic lifestyles of fungi [1].

Mycelia comprise of branching hyphal cells that extend at their apices. The apex represents the sink for the vectorial secretion of secretory vesicles generated within the hyphal network and is also the site of endocytosis. These vesicles provide membrane for tip expansion, wall matrix glycoproteins and biosynthetic enzymes for the assembly of the chitin and glucan wall skeleton. Vesicles are delivered to the apical surface in two stages: firstly via cytoplasmic transport, mediated mainly by microtubules, to a vesicle supply centre near the apex called the Spitzenkörper (apical body) [2^•^,3]; subsequently, by transport to the surface plasma-membrane mediated by the ‘Arp2/3 complex’, which organises apical actin [4], and an ‘exocyst complex’ which is responsible for vesicle docking and fusion [5,6]. The hyphal dome is rich in filipin-positive sterols that form a lipid-raft microdomain [7^•^,8,9]. A further group of apical proteins called the ‘polarisome’ have been defined in yeast and are required to mark and then polarise sites of growth. These include the Bud1 Ras-GTPase and Cdc42 Rho-GTPase modules which are responsible for recruiting actin and other components to the cell apex. Hyphal tip growth, therefore, involves the secretory pathway, cytoskeleton function and the activities of multimeric protein complexes that establish and maintain polarity. Detailed descriptions of this integrated process is beyond the scope of this article but have been reviewed elsewhere [2^•^,3,6,10–12,13^•^].

## Tropisms in plant pathogens and saprophytes

Tropic alignment of hyphae plays both general and specific roles in the growth of mycelial fungi. The hyphae within a mycelium exhibit avoiding reactions to each other resulting in the ramification of evenly spaced cells to maximise the occupation of substrate. The mechanism underpinning this negative autotropism has been hypothesised to be mediated by aerotropism towards oxygen, (away from oxygen-depleted zones around metabolically active hyphae) or negative chemotropism (away from staling products) but proof of either remains elusive. Hyphae can also fuse with one another, tip-to-tip or tip-to-hyphal side. A recent example of this behaviour has been in the discovery of conidial anastomosis tubes (CATs) in *Neurospora crassa* and other fungi. The physiological role of CATs is not yet known but they represent a common autotropism [14,15]. CAT signalling clearly involves some, as yet unknown, chemical signalling system that relays information between hyphae. A well-established tropic mechanism in relation to chemical signals in the environment is in the mating reactions between gametes of fungi [16,17]. However, evidence for tropisms in relation to chemical gradients (chemotropism) is remarkably sparse in most classes of fungi [18]. Chemotropism in ascomycetes has been described in the plant pathogen *Cochliobolus sativus* which grows towards, then infects, barley roots [19], and it is well known in the mycelial oomycetes, which are not fungi. Chemotropism has also been demonstrated in the rhizoids of certain zygomycetes such as *Allomyces macrogynus* [20], but, in general, evidence for fungal chemotropic orientation outside that related to sex pheromone responses is very limited.

**Figure 1:**
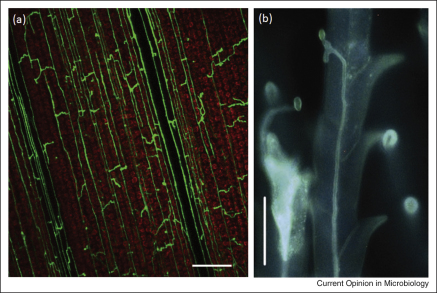
Tropic growth of fungi within plant tissues. **(a)** Intercalary hyphal extension by *Epichloë* endophytes in elongating grass leaves [21^•^]. Bar represents 100 μm. (Courtesy of C Voisey.) **(b)** Fluorescence microscopy of *C. purpurea* hyphae growing within a stigmatic hair towards the ovary of a rye plant [22]. Bar represents 20 μm. (Courtesy of P Tudzynski, with permission from Blackwell.)

Some of the most remarkable examples of tropic responses of hyphae are exhibited by plant pathogens and endophytes. Endophytic fungi form intimate non-pathogenic associations within plant tissues that require them to navigate around plant cortical cells and to coordinate their growth as plant tissues expand in meristematic regions (Figure 1a). This requires hyphae to exhibit intercalary extension — a phenomenon that runs against the dogma of polarised apical development of hyphae [21^•^]. Another example of a plant-directed tropism is in the invasion of rye and other grasses by germ tubes of the ergot fungus *Claviceps purpurea* where hyphal trajectories must be carefully orientated to traverse the length of the style to reach the region of the ovules (Figure 1b) [22]. *Uromyces* and *Puccinia* species and many other plant pathogens gain access to the plant by forming appressoria over the guard cells of stoma. The germ tubes of these fungi exhibit contact sensing and orientation in relation to the topography of the epithelial substratum (thigmotropism). Initially, hyphae grow perpendicularly across the plane of axis of the epithelial cells — a strategy that is thought to aid the location of stoma, which in monocotyledons are often positioned in staggered rows. When the germ tube encounters a guard cell of a specific lip height, an appressorium is induced (thigmodifferentiation) [23]. Elegant experiments with chemically inert plastic replica surfaces demonstrated that these events are mediated entirely by the topography of the plant surface and not by any chemical gradients [23–25]. In dicotylenous plants, the stoma occur within a mosaic of cortical cells on the plant surface and certain fungi follow the interstices between cells in order to locate them. In *Uromyces*, stretch-activated mechanosensitive channels have been studied [26] that may act as transducers of topographical information for thigmotropic growth (see below).

## Tropisms in human pathogens

**Figure 2:**
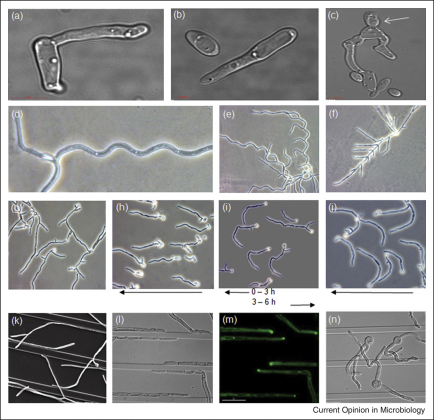
The tropic responses of *C. albicans*. **(a)** and **(b)** Chemotropism of mating projections (shmoos) in a gradient of mating pheromone. **(c)** Shmoo tips fusing to produce a daughter cell (arrow) (images from M Yang and N Gow). **(d)** and **(e)** Sinusoidal or helical growth induced by growth on semi-solid media. **(f)** Lack of sinusoidal growth when calcium homeostasis is disrupted by the deletion of the intracellular Ca^2+^ ATPase, Pmr1 [36]. **(g)**–**(j)** Galvanotropism of germ tubes of *C. albicans.* Hyphae are oriented randomly in controls (g) but are cathodally orientated in an applied electric field **(**h**)** (direction of the cathode is shown by arrows). If the field polarity is reversed after the establishment of cathodal growth, tips re-orient towards the new cathode position (i). Germ tube emergence towards the cathode is attenuated when the voltage-activated Ca^2+^ channel, Cch1, is deleted (j) [32^••^]. **(k)**–**(n)** Thigmotropism of *C. albicans.* Hyphae sense obstacles such as ridges in the substratum and re-orient tip growth (k) and (l). The concentration of the Rho-GTPase, Cdc42 (labelled with GFP), is tip-high (m) (images from A Brand). Deletion of Bud2, the GTPase-activating protein for the Ras-GTPase, Rsr1/Bud1, results in hyphae that are insensitive to ridges in the substrate (n) [29^••^]. For scale, the hyphal diameter is 2 μm in all photographs.

Tropic growth of hyphae of fungal pathogens has been reported for mating interactions for those few species that have recognised sexual cycles (Figure 2) [16,17]. Hyphal aerotropism has also been reported for *C. albicans* [27] which may be relevant in the relatively microaerophilic or anaerobic environments within a diseased tissue. The role of tropic hyphal growth in the penetration of mammalian tissue has been debated [28] but recent work has shown that *C. albicans* mutants that are compromised in tropic orientation are also attenuated in their ability to penetrate and damage epithelial cell layers [29^••^]. This lack of penetration also correlated with reduced virulence in an *in vivo* model of systemic infection, suggesting that normal regulation of tip orientation is required for penetrative growth. Thigmotropic responses of *C. albicans* and dermatophytes have also been described [29^••^,30,31,32^••^]. It has been suggested that some dermatophytes are negatively phototropic [33]. *Trichophyton metagrophytes* hyphae penetrate through the stratum corneum to locate and colonise hair follicles [34], but the sensing mechanism by which this targeting is achieved is not known.

Sinusoidal and helical growth of hyphae of *C. albicans* hyphae have also been described as a response to growth on hard or semi-solid surfaces [35,36]. Hyphae of *C. albicans* have also been shown to be highly orientated in applied electrical fields [32^••^,37]. The galvanotropic, thigmotropic and sinusoidal orientation responses of *C. albicans* have been exploited to examine the mechanisms underlying the regulation of tropic responses.

## Molecular mechanisms

Whilst the response to environmental cues is likely to be fungus-specific, tip re-orientation may be achieved by the modulation of the conserved machinery that sustains polarised hyphal growth. Recent studies in *C. albicans* and in *Aspergillus nidulans* have shown that mutants and experimental conditions can be devised that attenuate cell orientation responses without affecting tip growth. Hyphae normally grow in reasonably straight trajectories; however, reports have shown circumstances in which hyphae meander or exhibit sinusoidal growth. For example, hyphae of the human pathogen *C. albicans* will form sinusoidal hyphae on semi-solid surfaces (Figure 2) [35,36]. This sinusoidal growth was attenuated when external Ca^2+^ was depleted or various Ca^2+^ transporters were deleted implicating a role for calcium signalling in the coordination of hypha orientation [36]. Meandering growth in mutants with affected microtubule organisation and polarised actin assembly have also been described (see below). These studies suggest that the mechanism of tip orientation is distinguishable from the mechanism that determines apical extension and cell polarity, and they point to important roles for calcium signalling, Ras-type GTPase signalling modules, microtubule plus end organisation and for sterol-rich lipid rafts in regulating hyphal organisation.

### *Candida albicans*

**Figure 3:**
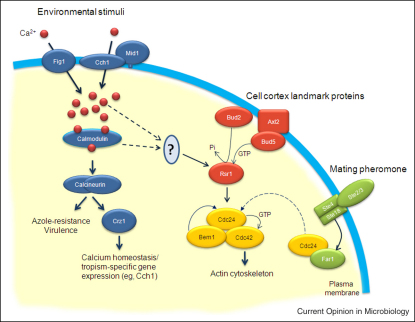
Directionality of growth is determined by at least three signalling pathways in *C. albicans*. During growth as yeast cells, bud site-selection is determined by interactions between cortical landmark proteins, including Axl2, adjacent to the site of previous bud outgrowth (axial budding), and Bud5, the guanine-exchange factor (GEF) for Rsr1/Bud1 [38]. During mating, the formation of polarised shmoos is directed by activation of the G-protein-coupled receptor, Ste2/3, by mating pheromone. Disassociation of the βγ subunits (Ste4 and Ste18) induces the release of Cdc24, the GEF for the Rho-GTPase, Cdc42 [56], from the Far1 complex. During multiple tropic responses, activation of plasma-membrane Ca^2+^ channels produces a localised increase in [Ca^2+^] which acts via unknown effectors on Rsr1/Bud1 to determine a new axis of hyphal growth.

Germ tube growth of *C. albicans* has been studied in some depth in terms of orientation mechanisms. In this fungus a variety of orientation responses have been shown to be dependent on calcium influx and homeostasis. *C. albicans* germ tube growth can be considered in terms of mechanisms that first establish polarity, then maintain it. The site of polarity in *Saccharomyces cerevisiae* is ploidy dependent and is determined by positional markers encoded by *BUD* genes that position buds at sites either adjacent to or opposite the site of bud scars (Figure 3) [38]. In *C. albicans*, however, *BUD* regulation of the site-selection mechanism of hyphae is relaxed and 50% of the evagination events are random [39,40]. Hypha orientation is, therefore, open to the influence of external cues (Figure 3). In adhered cells, emerging germ tubes always grow laterally from the mother cell, suggesting that the cell–substrate interface is sensed [41^•^].

The site of emergence can be influenced by the application of an external electric field [42], which, in *C. albicans*, causes germ tubes to emerge towards the cathode (Figure 2) [37]. The electric field is thought to depolarise the plasma-membrane on the cathodal face of the mother cell leading to activation of the voltage-gated Ca^2+^ channel, Cch1, and the elevation of tip Ca^2+^ in the hyphal apex. Localised Ca^2+^ gradients predict the site of outgrowth in diverse organisms, such as the brown alga, *Pelvetia compressa*, and pollen tubes [43,44]. Electrical fields may therefore orient germ tubes by inducing Ca^2+^ influx via Cch1. In support of this, deletion or blockage of Cch1, but not other Ca^2+^ channels, severely attenuates galvanotropism of *C. albicans* [32^••^] and other filamentous fungi [45]. Reciprocally, the galvanotropic response is enhanced in media with high extracellular [Ca^2+^]. Like galvanotropism, thigmotropism is attenuated by decreased Ca^2+^ availability. Deletion of Cch1 or the other two plasma-membrane Ca^2+^ channels, Fig1 or Mid1 (a mechanosensor that activates calcium influx via Cch1), reduces the sensitivity of hyphal tips to topographical features in the substratum. These observations suggest that the activation properties of specific plasma-membrane calcium channels may directly link environment-sensing with the generation of a localised Ca^2+^ signal that determines the site of tip growth, and therefore, orientation [32^••^,46].

Fungal tips are increasingly insensitive to small obstacles in the substrate as the angle of approach steepens [47,48]. It has been suggested that touch-sensing may not operate efficiently at the extreme apex of the apical dome due to its semi-fluid nature [48]. However, thigmotropic hyphae are tightly pressed to the substratum and typically have a nose-down morphology [48,49]. Since mechanosensitive signals must ultimately be translated into changes in the site of vesicle fusion in order to influence thigmotropic hypha orientation, it seems most likely that the tip orientation machinery must also reside within the apical dome.

The signalling pathway that links Ca^2+^ influx with tip re-orientation mechanisms (Figure 3) have not been established but proteins involved in polarity establishment are required for tropic responses. Deletion of the Ras-GTPase, Rsr1/Bud1, or its GTPase activating protein (GAP), Bud2, in *C. albicans* resulted in hyphae that could sustain polarised extension but were erratic in tip orientation. These signalling proteins ultimately serve to localise the recruitment of actin, and therefore, the vectorial secretion of vesicles to points of growth. Both mutants were unresponsive to external stimuli (Figure 2) [32^••^]. Real-time imaging of the *bud2*Δ mutant transformed with a green fluorescent protein (GFP)-tagged polarisome protein, Spa2, suggested that during hyphal growth the polarisome was not stabilised within the hyphal apex [50] so external signals may act by regulating the localisation of Rsr1 and members of the polarisome complex. In *S. cerevisiae*, the mating pathway requires Cdc42 but not Rsr1. Ultimately, it seems likely that there may be core elements that translate signals concerned with vegetative hyphal growth, bud site selection and the growth of mating projections of *C. albicans* into cellular orientation responses (Figure 3).

### *Aspergillus nidulans*

In obligatory filamentous fungi, a more significant role for microtubules emerges in long distance transport of secretory vesicles [13^•^,51]. Also, as in the fission yeast *Schizosaccharomyces pombe* and in *Aspergillus nidulans*, microtubules play important roles in the regulation of cell polarity [13^•^]. Zig-zag or meandering hyphal growth has been observed in strains that lack microtubule-associated kinesin motor proteins or the TeaA or TeaR cell end markers that localise actin nucleation to sterol-rich apical membranes [52^••^,53]. Tea proteins are also involved in the recruitment of formin proteins, such as SepA, that catalyse the polarisation of the actin cytoskeleton at the apex. Deletion of the kinesins, Kin1 in *Nectria haematococca* or KipA in *Aspergillus nidulans*, alter the central positioning or size of the Spitzenkörper and microtubules within the hypha fail to converge at a central point within the apex [54,55]. Microtubules and KipA were required for TeaA localisation at the hyphal apex and *teaA* and *kipA* mutants were affected in microtubule convergence at the hyphal apex [52^••^]. TeaA and TeaC, another cell-end marker protein, interact with the SepA formin protein. TeaC requires microtubules, but not KipA, for its localisation at hyphal tips and at septa [53]. Mutants in TeaC also exhibited zig-zag growth and were affected in septation. Therefore, the TeaA and TeaC proteins couple the microtubule and actin-based vesicle delivery systems involved in hypha orientation.

The Tea proteins and other apical membrane proteins within the polarisome complex reside within a sterol-rich domain that has been termed a ‘lipid-raft’ [7^•^8,9,52^••^]. Moderate to high concentrations of the sterol-binding dye filipin disrupted TeaR, and therefore indirectly, TeaA localisation [52^••^]. However, under these conditions zig-zag growth did not occur, suggesting that disruption of the apical sterol-rich domain had multiple effects on hyphal polarity.

**Figure 4:**
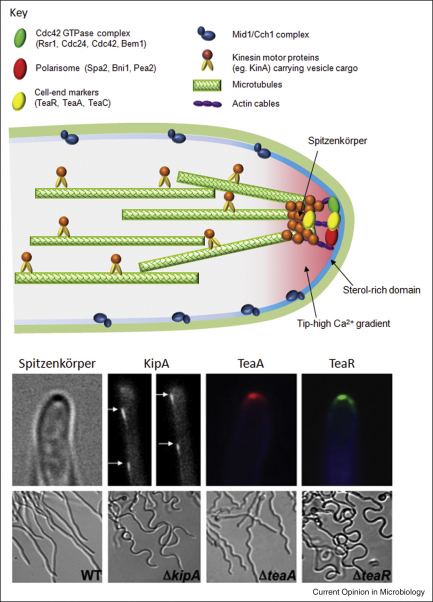
Components of the hyphal apex involved in orientation of the growth axis. Directionality involves calcium signalling through the Mid1–Cch1 channel complex, GTP–GDP cycling of the Ras-GTPase, Rsr1, delivery of specific cargo by kinesin motor proteins and proteins that tether microtubule plus ends to complexes in the apical sterol-rich domain. The Spitzenkörper is a vesicle assemblage fed by cytoplasmic transport of vesicles along microtubules which are tethered by via cell-end marker proteins. Vesicles finally are delivered to the membrane in the apex via actinomyosin. The spatial localisation of the KipA kinesin and Tea cell-end proteins in *A. nidulans* is shown along with the meandering mutant phenotypes of hyphae lacking these proteins (from R Fischer, with permission from Blackwell).

The control of directionality in hyphae, therefore, implicates both microtubules in delivering vesicles and localising parts of the actin-dependent vesicle docking system and localised calcium ion uptake and signalling via Ras-type GTPase modules that form part of the polarisome complex within a sterol-rich apical domain (Figure 4).

## Conclusions

The ability to orient the axis of growth of hyphae is a vital aspect of the physiology of fungal cells. Recent work has demonstrated that the molecular machinery that regulates hypha orientation can be distinguished from that inducing polarised growth since mutants and growth conditions can be generated, in which the trajectory of fungal hyphae is influenced without blocking the ability of hyphae to undergo apical growth. This orientation apparatus apparently involves calcium signalling, GTPase signalling modules and protein complexes that orchestrate sites of actin recruitment and microtubule-tethering at the hyphal apex. These components orchestrate the growth, morphogenesis and various lifestyles of filamentous fungi.

## References and recommended reading

Papers of particular interest, published within the period of review, have been highlighted as:

• of special interest
•• of outstanding interest
